# Microsatellite Instability in Pediatric High Grade Glioma Is Associated with Genomic Profile and Differential Target Gene Inactivation

**DOI:** 10.1371/journal.pone.0020588

**Published:** 2011-05-26

**Authors:** Marta Viana-Pereira, Alicia Lee, Sergey Popov, Dorine A. Bax, Safa Al-Sarraj, Leslie R. Bridges, João N. Stávale, Darren Hargrave, Chris Jones, Rui M. Reis

**Affiliations:** 1 Life and Health Sciences Research Institute (ICVS), School of Health Sciences, University of Minho, Braga, Portugal; 2 Section of Paediatric Oncology, Institute of Cancer Research, Sutton, Surrey, United Kingdom; 3 Paediatric Oncology, Royal Marsden Hospital, Sutton, Surrey, United Kingdom; 4 Department of Clinical Neuropathology, Kings College Hospital, London, United Kingdom; 5 Neuropathology, St George's Hospital, London, United Kingdom; 6 Department of Pathology, Federal University of São Paulo, São Paulo, Brazil; 7 Molecular Oncology Research Center, Barretos Cancer Hospital, Barretos, Brazil; University Medical Center Hamburg-Eppendorf, Germany

## Abstract

High grade gliomas (HGG) are one of the leading causes of cancer-related deaths in children, and there is increasing evidence that pediatric HGG may harbor distinct molecular characteristics compared to adult tumors. We have sought to clarify the role of microsatellite instability (MSI) in pediatric *versus* adult HGG. MSI status was determined in 144 patients (71 pediatric and 73 adults) using a well established panel of five quasimonomorphic mononucleotide repeat markers. Expression of MLH1, MSH2, MSH6 and PMS2 was determined by immunohistochemistry, *MLH1* was assessed for mutations by direct sequencing and promoter methylation using MS-PCR. DNA copy number profiles were derived using array CGH, and mutations in eighteen MSI target genes studied by multiplex PCR and genotyping. MSI was found in 14/71 (19.7%) pediatric cases, significantly more than observed in adults (5/73, 6.8%; p = 0.02, Chi-square test). MLH1 expression was downregulated in 10/13 cases, however no mutations or promoter methylation were found. MSH6 was absent in one pediatric MSI-High tumor, consistent with an inherited mismatch repair deficiency associated with germline *MSH6* mutation. MSI was classed as Type A, and associated with a remarkably stable genomic profile. Of the eighteen classic MSI target genes, we identified mutations only in *MSH6* and *DNAPKcs* and described a polymorphism in *MRE11* without apparent functional consequences in DNA double strand break detection and repair. This study thus provides evidence for a potential novel molecular pathway in a proportion of gliomas associated with the presence of MSI.

## Introduction

Pediatric gliomas comprise a diverse group of lesions which account for more than half of all childhood CNS tumors. In contrast to adults, where WHO grade IV glioblastomas predominate, in children the most common form are WHO grade I pilocytic astrocytomas [1]. Despite the rarity of high grade gliomas (HGG) in children, they are one of the leading causes of cancer-related deaths in this age group, with a current two year survival rate of 10–15% [2]. Although histologically similar to those which arise in adults, HGG in children may have distinct clinical features, including anatomical site of presentation [2] and response to chemotherapy [3].

New evidence also demonstrates that pediatric HGG harbor distinct genetic characteristics compared to adult tumors. Recent large-scale genomic studies on adult glioblastomas defined the key genetic aberrations, and proposed the ‘core signaling pathways’ driving gliomagenesis [4], [5]. Similar studies on pediatric tumors demonstrated fewer genomic events targeting these pathways, and identified alterations in PDGF-driven signaling to be prevalent in the majority of pediatric tumors, in contrast to adults, where EGFR is the predominant target [6]. The finding of a significant proportion of childhood HGG to harbor few chromosomal imbalances is one key difference to those seen in adults [6], and raises questions regarding the underlying biological basis for these highly aggressive tumors.

The presence of microsatellite instability (MSI), is another feature that has been described to be more frequent in pediatric than in adult brain tumors [7], [8], [9], [10], [11]. Nevertheless, the results are conflicting, with reported frequencies in pediatric gliomas varying between 0–44%, possibly due to different sensitivities of the methods used to detect MSI status [12], [13], [14], [15], [7], [8], [9], [10], [11], [16], [17]. MSI was first described and is better characterized in hereditary non-polyposis colorectal cancer (HNPCC, Lynch syndrome) [18], where it is thought to arise due to germline mutations in mismatch repair (MMR) genes, mainly in *MLH1*, *MSH2*, *MSH6* and *PMS2* [19]. Germline mutations in these genes have also been described in Turcot's syndrome, which predispose to gliomas [1].

Both in familial and sporadic colorectal cancer (CRC), the presence of MSI is associated with mutations in genes harboring microsatellites in their coding or regulatory regions. Genes involved in DNA repair, cell growth inhibition and apoptosis are targeted, although the frequency varies between cancers [20], and an extensive analysis has not been previously performed in MSI gliomas. We have sought to clarify the role of MSI in pediatric *versus* adult HGG, and report a higher prevalence of MSI in childhood cases. We have further investigated the role of MMR system proteins, genomic instability and 18 known target genes in pediatric MSI HGG.

## Methods

### Cases and DNA isolation

Formalin-fixed paraffin-embedded (FFPE) HGG samples from 71 children and young people (<23 years) and 73 adults (32–79 years) were obtained after approval by Local and Multicenter Ethical Review Committees from King's College Hospital and St George's Hospital, UK and Federal University of São Paulo, Brazil (Table S1). The pediatric cases were obtained from all three Health Institutions. All the samples enrolled in the present study were unlinked and unidentified from their donors. Due the retrospective nature of the study, no written informed consent from patients was obtained, with the exception of the UK samples obtained after 2006, where all patients signed a written informed consent, following the UK Human Tissue Act approved in that year. The Local Ethical Review Committees of Federal University of São Paulo, Brazil waived the need for written informed consent. The presence of tumor tissue in these samples and the tumor histology was verified on a H&E-stained section independently by three neuropathologists (SA-S, LRB, JNS). DNA was isolated using the QIAamp DNAMini kit (Qiagen) according to the manufacturer's instructions.

### MSI analysis

MSI was assessed using a multiplex PCR comprising five quasimonomorphic mononucleotide repeat markers (NR27, NR21, NR24, BAT25 and BAT26), as previously described [21]. Products were separated using an ABI Prism 3100 genetic analyzer (Applied Biosystems) and results analyzed with GeneScan Analysis software, version 3.7 (Applied Biosystems). In the absence of matched normal DNA, MSI was defined as MSI-High (MSI-H, instability at three or more markers) MSI-Low (MSI-L, instability at one or two markers) or microsatellite stable (MSS, absence of instability) [22]. The quasimonomorphic variation range (QMVR) of each marker, previously described [23] was established in our analysis using a series of DNA samples from 30 controls which were randomly selected from cancer-free blood donors at the ICR, UK and São Marcos Hospital, Portugal.

### Mutation analysis of MSI-targeted genes

Selected genes containing repeat sequences, previously described as frequent targets for MSI in other cancers [24], [25], [26], were chosen for mutation screening (primers available upon request): *ATM* (poly(T)13), *ATR* (poly(A)10), *AXIN2* (poly(G)7; poly(C)5; poly(C)6), *BAX* (poly(G)8), *BLM* (poly(A)9), *BRCA1* (poly(A)8), *BRCA2* (poly(A)8), *DNAPKcs* (poly(A)10), *MBD4* (poly(A)10), *MRE11* (poly(T)11), *MSH3* (poly(A)8), *MSH6* (poly(C)8), *PTEN* exon 7 (poly(A)6), *PTEN* exon 8 (poly(A)6), *RAD50* (poly(A)9), *TCF4* (poly(A)9), *TGFβRII* (poly(A)10), *WISP3* (poly(A)9) and *XRCC2* (poly(T)8). PCR and genotype analysis were performed as previously described [21], except *AXIN2*, which was directly sequenced after PCR [25]. Samples presenting abnormal profiles were direct sequenced to confirm the presence of frameshift mutations.

### Immunohistochemistry

Immunohistochemistry for MLH1, MSH2, MSH6, PMS2 and MRE11 was performed using the Vectastain ABC system (Vector), according to the manufacturer's instructions. Antigen retrieval was achieved in boiling waterbath in Tris-EDTA pH 9.0 for 20 min and primary antibodies MLH1 (G168-15, 1∶25, BD Biosciences), MSH2 (FE11, 1∶150, Calbiochem), MSH6 (44, 1∶100, BD Biosciences), PMS2 (A16-4, 1∶200, BD Biosciences) and MRE11 (12D7, 1∶500, Abcam) were incubated overnight at 4°C.

For DNAPKcs staining, the Ultravision Plus Detection System (LabVision) was used according to the manufacturer's instructions. Antigen retrieval was performed with microwave treatment in citrate buffer pH 6.0 for 15 min and primary antibody (Ab-4 cocktail, 1∶100, NeoMarkers) incubated 2 h at RT.

Microscopic analysis was done by a blinded pathologist (S. P.). Sections without staining in the tumor cells were considered to have a lost expression (−). Samples without nuclear staining but positivity in the cytoplasm or with <5% tumor cells with nuclear staining were considered with diminished expression (+). Samples with the scores ++ (5–50% nuclear staining in tumor cells) or +++ (>50% nuclear staining in tumor cells) were considered positive.

### Mutation analysis and methylation-specific (MS) - PCR of *MLH1*

Mutation analysis of *MLH1*, exons 1 to 19, was performed by PCR, using primers previously described [27], followed by direct sequencing.

For *MLH1* promoter methylation detection, DNA was treated with sodium bisulphite using the Epitect kit (Qiagen) according to the manufacturer's instructions. MS-PCR was performed using primers previously described [28].

### Cell culture and ionizing irradiation

The cell lines Daudi, Raji and Jurkat were kindly provided by Dr Sue Colman/Prof Mel Greaves (ICR, UK). Cells were grown in suspension in RPMI 1640 medium supplemented with 10% fetal bovine serum at 37°C in 5% CO_2_. Cells were seeded in 35 mm Petri dishes and treated with 1.5 Gy ionizing irradiation (IR) using an X-ray source irradiator (HS-MP1, AGO) operating at 250 kv and 10 mA. Cells were allowed to recover for 1 h, 4 h or 24 h and then processed for immunofluorescence staining or western-blot analysis.

### Immunofluorescence

Cells were spun at 200 rpm for 5 min on a Shandon Cytospin 3 centrifuge (Thermo Fisher Scientific), fixed according to a modified Strecks pre-extraction protocol, permeabilized and blocked, as described [29]. Primary antibodies directed against MRE11 (Sheep polyclonal, 1∶500, previously described [30]), 53BP1 (NB100-304, rabbit polyclonal, 1∶100, Novus Biologicals) and phospho-Histone H2A.X (Ser139) (JBW301, mouse monoclonal, 1∶2000, Millipore) were applied overnight at 4°C and secondary antibodies, Alexa Fluor 594 donkey anti-goat IgG, Alexa Fluor 488 donkey anti-rabbit IgG and Alexa Fluor 488 donkey anti-mouse IgG (Invitrogen) 1 h at RT. Cell nuclei were stained with DAPI (Invitrogen). Images were taken using a Zeiss Axioplan 2 microscope and Smartcapture 2 software (Digital Scientific).

### Western blot analysis

Protein extracts were separated in a 3–8% Tris-Acetate gel (Invitrogen) and transferred to PVDF membranes (GE Healthcare). Immunodetection was performed using antibodies directed against phospho-ATM (Ser1981) (10H11.E12, 1∶500, Cell Signaling), ATM (MAT3-4G10/8, 1∶1000, Sigma-Aldrich), and MRE11 (12D7, 3 µg/mL, Abcam).

### Quantitative Real-Time PCR

cDNA was prepared from 1 µg of RNA by random primed reverse transcription using Superscript III (Invitrogen). qRT-PCR was performed using SYBR green master mix (Applied Biosystems) on an ABI 7900HT loaded with the SDS2.1 software. Primers used were as follows: total MRE11 mRNA (exons 2–4), CCAGGGGTTCTTGGAGAAG (forward) and TTTCCTTGAGGGCTTATTTTCA (reverse); MRE11 distinguishing the aberrant and wild-type transcript (exons 2–6) included the same forward primer and CCAGCACAACTTAAAATGTC (reverse); GAPDH, GCCACCCAGAAGACTGTGGATGGC (forward) and CATGATGGCCATGAGGTCCACCAC (reverse). GAPDH mRNA levels were measured as an internal control. The number of amplification cycles to half maximal saturation of the PCR product was determined by measuring the integration of the fluorescent dye into the PCR products. The ratio of the level of MRE11 expression relative to the GAPDH control was calculated. Samples were analyzed on a 4% agarose gel loaded after saturation of the PCR reaction.

### Statistical Analysis

All statistical tests were done in SPSSv16.0 (SPSS Inc.). Correlations between categorical values were done using the two-tailed Chi-square and Fisher's exact tests. A p value of <0.05 was considered significant.

## Results

### MSI is more common in pediatric HGG than in adults

**Figure 1:**
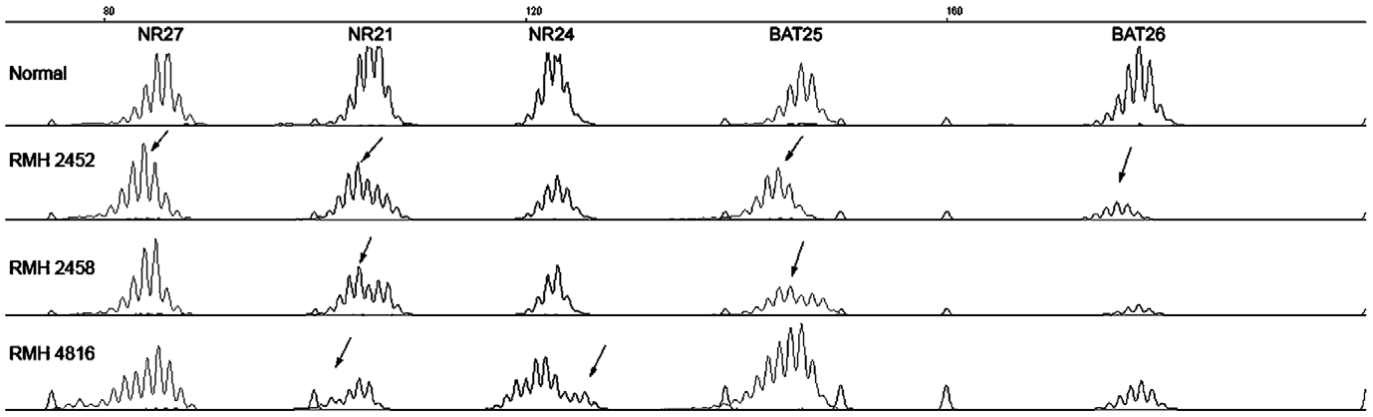
Microsatellite instability in pediatric HGG. Representative electropherogram traces for three MSI-positive pediatric HGG, presenting sequence alterations in more than one quasimonomorphic marker. Case RMH2452 (MSI-H) presented alterations in NR27, NR21, BAT25 and BAT26; RMH2458 (MSI-L) in NR21 and BAT25; and RMH4816 (MSI-L) in NR21 and NR24. Alterations in relation to a control trace are indicated with arrows.

MSI analysis was performed in 144 HGG, 71 pediatric and 73 adults, using a pentaplex PCR of quasimonomorphic markers as recommended by the revised Bethesda guidelines [18]. A total of 19 samples (13.2%) presented instability, 1 MSI-H (<1%) and 18 MSI-L (12.5%) cases, with the remaining 125 tumors stable (86.8%). The MSI-H case, RMH2452, was a three year old girl with glioblastoma, presenting instability at four markers (NR27, NR21, BAT25 and BAT26) (Figure 1). Overall, there were 14/71 (19.7%) MSI-positive pediatric cases, significantly more than observed in adults (5/73, 6.8%; p = 0.02, Chi-square test). The pediatric MSI cases comprised 11 glioblastomas, two anaplastic astrocytomas and one anaplastic oligodendroglioma, with an age range of between 4 months and 20 years. The adult MSI-L cases were glioblastomas of ages 62–75 years (Table 1). Individual microsatellite data for each case is provided in full in Table S1.

**Table 1:** Clinical characterization and MMR status of MSI cases.

*Case*	*Gender/Ag e*	*WHO grade*	*Diagnosis*	*MSI status*	*Immunohistochemistry*				*MLH1 mutation screening*	*MLH1 methylation status*
					*MLH1*	*MSH2*	*MSH6*	*PMS2*		
**Pediatric**										
*2452*	*F/3*	*IV*	*GBM*	*MSI-H*	*+++*	*+++*	*−*	*++*	*ND*	*ND*
2458	F/9	IV	GBM	MSI-L	+++	+++	+++	++	A	A
4816	M/14	IV	GBM	MSI-L	+	++	++	++	A	NA
2444	M/19	IV	GBM	MSI-L	−/+	+++	+/++	++	A	A
2457	F/13	IV	GBM	MSI-L	−/+	++	+++	−/+	A	A
2470	F/14	IV	GBM	MSI-L	MC	MC	+/++	MC	A	A
3952	F/14	IV	GBM	MSI-L	−	NS	NA	++	NA	NA
3954	M/20	IV	GBM	MSI-L	+++	++	+++	+++	A	A
3962	F/0.3	III	AA	MSI-L	+	−	++/+++	−/+	A	A
3967	F/8	III	AO	MSI-L	+	++	++/+++	NS	A	A
3969	F/14	IV	GBM	MSI-L	MC	MC	+/++	MC	A	A
4820	M/16	IV	GBM	MSI-L	+	+	+	++	NA	NA
4823	M/15	IV	GBM	MSI-L	+	++	−	++	NA	NA
4839	M/16	IV	GBM	MSI-L	NS	++	++	++	NA	NA
**Adult**										
3248	M/67	IV	GBM	MSI-L	+++	+++	+++	++	A	NA
3283	M/63	IV	GBM	MSI-L	+++	++	++/+++	+++	A	NA
3415	M/70	IV	GBM	MSI-L	NA	NA	NA	NA	NA	NA
3416	F/75	IV	GBM	MSI-L	−	+++	+++	+++	A	NA
3465	F/62	IV	GBM	MSI-L	NS	+++	+++	+++	A	NA

Abbreviations: M, male; F, female; GBM, glioblastoma; AA, anaplastic astrocytoma; AO, anaplastic oligodendroglioma; MSI-H, microsatellite instability-High; MSI-L, microsatellite instability-Low; MC, mostly cytoplasmatic; NS, non-specific; A, absent; ND, not done; NA, not available.

### Inactivation of mismatch repair proteins in familial and sporadic MSI pediatric HGG

Aiming to determine the underlying nature of the MSI observed we sought to investigate whether key components of the MMR system were intact, screening the MSI-positive samples for expression of MLH1, MSH2, MSH6 and PMS2 by immunohistochemistry (Table 1, Figure S1). 10/13 (76.9%) pediatric samples with MSI showed an absent or diminished expression of MLH1 in the tumor cells, often in concert with reduced MSH2 (4/10, 40%) or PMS2 expression (4/10, 40%). There was, however, no evidence of mutation (assessed by direct sequencing) or hypermethylation (by MS-PCR) of *MLH1* in these cases. Due to the FFPE nature of the samples, it was not possible to screen for *MLH1* mutations and promoter methylation in all MSI samples (Table 1). Moreover, on samples screened for *MLH1* mutations, the rate of successful exons sequenced ranged from 58% (11/19 exons) to 100% (19/19 exons), according sample DNA quality. In contrast to pediatric, the four adult MSI-L cases presented positive immunoreactivity for MMR proteins.

A single case was negative only for MSH6. This patient (RMH2452, MSI-H) presented multiple café-au-lait spots in the absence of other clinical features of neurofibromatosis-1 (NF1). There was no family history of NF1, although the maternal great grandmother had endometrial cancer in her early forties. Although constitutional DNA was not available for testing, this patient's clinical history is consistent with an inherited MMR deficiency such as Turcot's syndrome, associated with germline *MSH6* mutation.

### MSI-positive pediatric HGG have distinct genomic profiles and differential target genes compared with other tumor types

**Figure 2:**
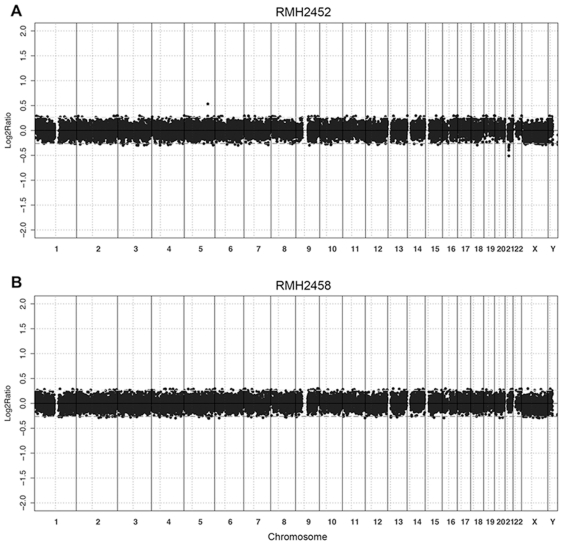
Genomic stability in MSI pediatric HGG. Array CGH genome plots are shown for two MSI-positive pediatric HGG, the MSI-H RMH2452 (A) and the MSI-L RMH2458 (B), demonstrating lack of large scale alterations detectable on the 32K BAC platform. Log_2_ ratios for each clone (y axis) are plotted according to chromosomal location (x axis). The centromeres are represented by vertical lines.

As the presence of MSI reflects a nucleotide-level form of genetic instability, work in CRC suggests that large scale chromosomal instability is reduced or absent in MSI-positive cases [31], [32]. In order to determine whether this may also be true for pediatric HGG, we examined copy number profiles on 9/14 MSI-positive cases for which sufficient quantity and quality of DNA was available, and compared this to a similarly profiled cohort of 26 pediatric MSS cases (Array Express accession number E-TABM-857) [33]. A total of four MSI-positive cases harbored a ‘flat’ or ‘stable’ profile, with minimal or no copy number alterations detectable on the 32K tiling-path BAC array platform used (Figure 2, Table 2). This is a pattern of genomic stability present in approximately 20% of pediatric HGG, but almost entirely absent from similar adult tumors [6], [34]. Four MSI-L cases harbored a small number of whole chromosome arm gains or losses, and fell into the ‘aneuploid’ category of genomic profile. There was a single, MSI-L, case (RMH3954) that had a highly rearranged genome, with 19 distinct alterations. No MSI-positive cases contained any high-level amplifications or homozygous deletions. Overall there were less copy number changes in MSI (mean 5.78, range 0–19) than MSS cases (mean 8.35, range 0–25), although there was no statistical difference in the groups (p = 0.37, t test), reflecting the highly rearranged MSI-L case, and the presence of 4/26 (15.4%) genomically stable cases by array CGH that were also MSS.

**Table 2:** Genomic alterations and Candidate MSI target genes frameshift mutations in MSI samples.

*Case*	*MSI status*	Genomic subtype	Copy number changes	Altered MSI Target Genes
**Pediatric**				
*2452*	*MSI-H*	*Stable*	*5q31+*, *21q21−*	*MSH6 (C)9*
2458	MSI-L	Stable	None	-
4816	MSI-L	NA	NA	-
2444	MSI-L	Aneuploid	1q+, 9q+	MRE11 (T)11/12
2457	MSI-L	Aneuploid	4q−, 5q−,7p12+, 9p21−, 13q−, 19+	-
2470	MSI-L	Aneuploid	5q−, 10q−, 14q−, 15q−, 19+, 20p−, Xp−	-
3952	MSI-L	Aneuploid	1p−, 6p−, 12q− 13q−, 19−, 22q−	-
3954	MSI-L	Rearranged	1p−, 1q+, 2q+, 3q−, 4p+, 4q+, 4q−, 5q−, 6p−, 6q+, 7+, 8p+, 8q−, 11p−, 11q+, 12q−, 13q−, 18−, 19q−	-
3962	MSI-L	Stable	None	-
3967	MSI-L	Stable	None	-
3969	MSI-L	NA	NA	DNAPKcs (A)9/10
4820	MSI-L	NA	NA	-
4823	MSI-L	NA	NA	-
4839	MSI-L	NA	NA	MRE11 (T)11/12
**Adult**				
3248	MSI-L	NA	NA	MRE11 (T)11/12
3283	MSI-L	NA	NA	-
3415	MSI-L	NA	NA	-
3416	MSI-L	NA	NA	-
3465	MSI-L	NA	NA	-

Abbreviations: MSI-H, microsatellite instability-High; MSI-L, microsatellite instability-Low; NA, not available.

The presence of MSI confers an increased susceptibility for acquiring mutations in various target genes containing single nucleotide repeat sequences [20]. Selected CRC target genes involved in apoptosis (*BAX*), tumor growth (*TGFβRII*), WNT pathway (*AXIN2*, *TCF4*, *WISP3*), DNA repair (*ATM*, *ATR*, *BLM*, *BRCA1*, *BRCA2*, *DNAPKcs*, *MBD4*, *MRE11*, *MSH3*, *MSH6*, *RAD50*, *XRCC2*) and PI3-kinase signaling (*PTEN*) were analyzed for mutations in MSI-positive HGG samples (Table 2).

**Figure 3:**
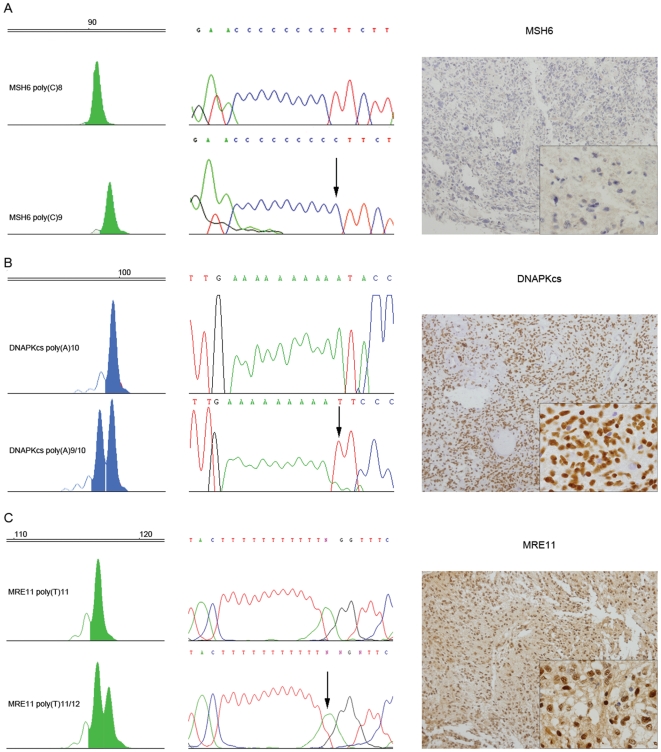
Target gene screening in MSI pediatric HGG. Electropherogram traces, direct sequencing, and immunohistochemistry for MSI target genes presenting frameshift mutations in pediatric HGG (DNA alterations indicated with arrows). (A) Case RMH2452 (MSI-H) presenting a homozygous insertion of one bp in the MSH6 poly(C)8 tract and loss of protein expression. (B) RMH3969 (MSI-L) presenting a heterozygous deletion of one bp in the DNAPKcs poly(A)10 tract, and retention of protein expression. (C) RMH2444 (MSI-L) presenting a heterozygous insertion of one bp in the MRE11 poly(T)11 tract and retention of protein expression.

The pediatric MSI-H sample was found to contain a homozygous single base insertion in the poly(C)8 tract of *MSH6* poly(C)9, a frameshift which results in a truncated protein, confirmed in duplicate by genotyping and direct sequencing (Figure 3A). Somatic mutations on the poly(C)8 sequence of *MSH6*, are strongly associated with initiating *MSH6* germline mutations [35], adding further evidence to this patient having an inherited MMR deficiency syndrome.

A further MSI-L sample (RMH3969) presented a heterozygous single base deletion in the poly(A)10 sequence on the exon 5 of *DNAPKcs* (poly(A)9/10), however this mutation did not influence protein expression, as observed by immunohistochemistry (Figure 3B). Two other MSI-L cases, RMH2444 (Figure 3C) and RMH3952 harbored a heterozygous single base insertion in the poly(T)11 tract on the intron 4 of *MRE11* (poly(T)11/12). Immunohistochemistry again revealed positivity of the protein (Figure 3C). This insertion was also observed in 1/5 adult cases with MSI-L (RMH3248). No additional target gene mutations were observed in our series (Table 2).

### MRE11 poly(T)11/12 is a previously unrecognized polymorphism with no apparent functional consequence in DNA double strand break detection and repair

**Figure 4:**
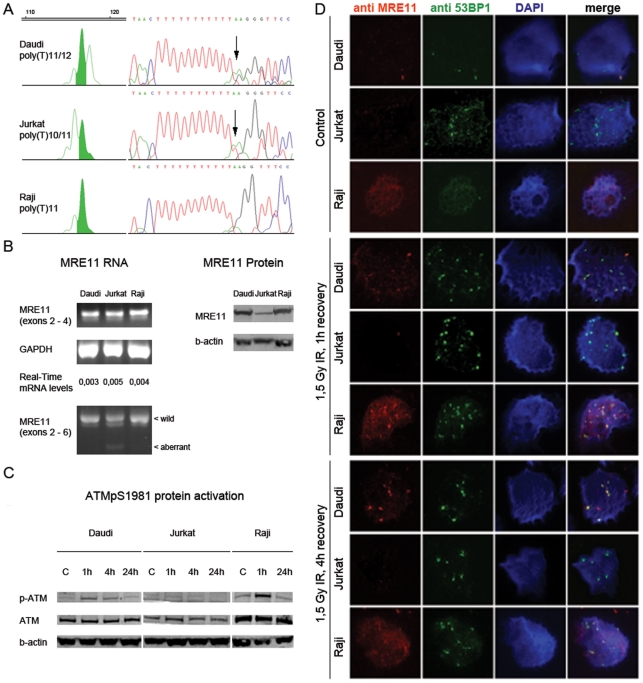
*MRE11 poly(T)11/12 sequence variant has no apparent functional* consequence on DNA damage detection and repair. (A) Electropherograms and sequencing traces of the MRE11 poly(T)11 tract in leukemia/lymphoma cells: Daudi (heterozygous insertion of one bp, poly(T)11/12); Jurkat (heterozygous deletion of one bp, poly(T)10/11); and Raji (wild-type, poly(T)11). (B) MRE11 expression in Daudi, Jurkat and Raji cells. qRT-PCR was used to calculate the ratio of MRE11 expression (amplification of exons 2 to 4) relative to the GAPDH control, independently of the presence of the aberrant transcript observed in Jurkat. PCR products were loaded on the gel at saturation of the reaction, being not quantitative. MRE11 protein levels were assessed by Western blot, and were considerably lower in Jurkat. (C) ATM-pS1981 levels were assessed by Western blot in Daudi, Jurkat and Raji after 1.5 Gy irradiation of cells and 1 h, 4 h or 24 h recovery. There was an increase of ATM phosphorylation 1 h after irradiation in Daudi and Raji cells that decreased at the later time points. Jurkat showed no ATM phosphorylation after exposure to irradiation. (D) DNA repair foci was observed by immunofluorescence after 1.5 Gy IR followed by 1 h and 4 h recovery. Irradiation resulted in the formation of both MRE11 and 53BP1 foci in Daudi and Raji cells, whereas Jurkat failed to form MRE11 foci. Nuclei were counterstained with DAPI. Merged figure presents co-localization of MRE11 and 53BP1 foci visualized in yellow.

As MRE11 represented a potentially unknown contributor to the pathogenesis of HGG associated with MSI, we determined whether the specific *MRE11* poly(T)11/12 mutation affects the normal function of the protein, a member of the MRE11-RAD50-NBS1 (MRN) complex involved in DNA repair. As models we used three leukemia/lymphoma cell lines (Figure 4A): Daudi, *MRE11* poly(T)11/12; Raji, *MRE11* poly(T)11/11, wild type, and DNA repair proficient; and Jurkat, *MRE11* poly(T)10/11 known to generate alternative splicing in exon 5 of *MRE11* (Figure 4B) and DNA repair deficient [26]. The presence of an aberrant transcript was detected by RT-PCR of exons 2 to 6with a truncated product detected in Jurkat cells (Figure 4B). Levels of total MRE11 mRNA were quantified by qRT-PCR amplification of exons 2 to 4 which generates a single size product, for both wild-type and the aberrant transcript. The ratios of the mRNA expression levels of MRE11, relative to GAPDH, were similar across all cell lines (Figure 4B) while protein levels were lower in Jurkat (Figure 4B).

To assess the proficiency of MRE11-associated DNA repair in *MRE11* poly(T)11/12 Daudi, we monitored the repair response following DNA damage induced by ionizing radiation. After exposure to irradiation, auto-phosphorylation of ATM on serine-1981 takes place in response to the formation of DNA double stranded breaks (DSB) [36]. 1.5 Gy IR resulted in no ATM phosphorylation in Jurkat, while Raji and Daudi, presented an increase of ATM phosphorylation 1 h after IR followed by a slight decrease in longer time points (Figure 4C). Another response to ionizing radiation is the accumulation of DNA repair proteins after exposure to irradiation that can be visualized in the form of foci associated with recruitment of essential repair complexes to the sites of DNA damage [37]. After 1.5 Gy IR, 53BP1 foci formation after IR was proficient in all cell lines as these can form even in the absence of a functional MRN complex (Figure 4D). In Daudi and Raji, the co-localization between MRE11 and 53BP1 foci observed was suggestive of the formation of functional repair complexes, in contrast to Jurkat, where no MRE11 foci were present. Compared to Jurkat and Daudi, Raji non-irradiated cells showed higher background levels of DNA damage as detected by formation of γH2AX foci (Figure S2). In summary, these results suggest that the *MRE11* poly(T)11/12 mutation does not result in a compromised DNA damage repair response in Daudi cells.

Although *MRE11* poly(T)11/12 alteration could not be found in the dbSNP database, this specific base change was recently identified in the germline of a Caucasian male [38]. We subsequently screened a series of 72 control DNA samples, and discovered *MRE11* poly(T)11/12 to be present in 5/72 (6.9%) healthy volunteers. Although this frequency is less than that observed in the MSI-positive cases (3/19, 15.8%), the difference is not statistically significant (p = 0.23, Chi-square test). Thus we conclude that *MRE11* poly(T)11/12 is a previously unrecognized polymorphism with no functional consequences on the DNA repair response.

## Discussion

Published studies on MSI in HGG have produced contradictory results. These differences have variously been attributed to the number of samples studied, the heterogeneity and accuracy of methods used to determine MSI status. We sought to address this by carrying out a large study of HGG using the most robust and sensitive screens available, and report a significantly elevated frequency of MSI in pediatric (19.7%) *versus* adult (6.8%) tumors.

One of the key considerations in the assessment of MSI is in the use of mononucleotides *versus* polynucleotides in the panel of markers used. The quasimonomorphic nature of the mononucleotides we used makes these most suitable for MSI detection, particularly when matching germline DNA is not available, and display a higher sensitivity [23], [39]. Many of the early studies used polynucleotide markers only and found a frequency of MSI ranging from of 0 to 37% in series of 7 to 56 gliomas from pediatric or age not discriminated series [12], [13], [14], [15]. Including both mononucleotide and polynucleotides did little to improve consistency, with contrasting results of 0 to 44% in small series of pediatric gliomas [8], [9], [10] and 4% MSI reported in a recent study using a larger series of 68 pediatric HGG [40]. Recent studies using mononucleotides only were also contradictory. Alonso *et al.* 2001 [11] reported a frequency of 27% MSI in 45 pediatric HGG using BAT25 and BAT26 as markers, while Eckert *et al.* 2007 [16] found no evidence of MSI in 41 cases using CAT25, BAT25 and BAT26. Vladimirova *et al.* 2007 [17] used the same panel of five mononucleotides as in the present study, and yet found a reduced frequency of 3.2% MSI in 126 pediatric HGG in comparison to our 19.7%. One major difference between our studies that possibly accounts for the diversity of frequencies is the use of 30 controls to establish the QMVR in our work. As recently demonstrated, this approach obviates the need for amplification of matched normal DNA to determine instability in the tumor tissue, and provides the most robust strategy to identify MSI tumors [39]. In addition, this approach allowed us to assess smaller allelic shifts of the markers as any variation outside the QMVR was considered as instability. Although the data presented by previous studies do not allow for re-analysis using this technique, we speculate that our increased frequency of MSI may be related to enhanced detection sensitivity [39]. In fact, a study in malignant gliomas, that used a panel of markers similar to the one used in our work and performed a QMVR using DNA from 7 normal controls, described the presence of MSI-L in 15% (8/52) of cases, a frequency comparable to our results (13%) [41].

Another important analytical difference may be found in the use of an alternative classification of MSI which takes into account the size of the allelic shifts in the markers, rather than the number of markers with alterations. In this classification, samples presenting small length change (≤6 bp) are considered Type A, whilst those presenting more drastic alterations are described as Type B MSI, as reported for CRC [42]. Pediatric HGG with constitutive MMR deficiency have been previously described as possessing Type A MSI [43] and we also observed Type A MSI in our sporadic, and likely syndromic cases. This is also consistent with our previous observations in medulloblastoma [21], and appears to be a consistent difference in MSI reported in CNS tumors compared with the classic MSI positive epithelial tumors such as colorectal and gastric carcinomas. The observation that brain tumors present smaller changes in the MSI markers may represent a cause of the inconsistency found on MSI frequencies reported in literature.

Having established that a proportion of our pediatric HGG cases harbored MSI, we were keen to determine how this related to other genomic abnormalities. Microsatellite and chromosomal instability have been considered to be mutually exclusive [31], and although recent reports have demonstrated that a minority of tumors can present both MSI-H and chromosomal instability, the frequency and degree of chromosomal alterations in colorectal and gastric MSS cancers is much higher than in the MSI-H cases [32]. Pediatric HGG differ from those found in adults by comprising a proportion of tumors with few or no detectable copy number changes by microarray analysis [6]. The MSI-H sample in our current cohort fell into that category. The MSI-L cases also had relatively fewer alterations than the population as a whole, while the presence of MSI and chromosomal instability were not mutually exclusive. In particular, there were several cases with ‘stable’ genomes which did not present MSI, suggesting that although this phenotype was associated with a proportion of cases with no gross alterations, it was not a general explanation for this key difference between the childhood and adult disease.

MSI is a molecular feature resulting mainly from inactivating alterations of the MMR system [19]. We studied MMR inactivation by immunohistochemistry on the 11 MSI tumors and observed that MLH1 expression was absent or reduced in 10 samples, 5 of which also presented reduced PMS2 and/or MSH2 expression. Considering the high percentage of pediatric HGG lacking MLH1 expression, molecular deficiencies in this gene appear to be the origin of the MSI phenotype in most of the cases, although we did not observe point mutations or hypermethylation in these samples.

MSH6 expression was absent in a single sample that also presented a homozygous insertion of a single nucleotide in the poly(C)8 track of *MSH6*, which is a target of MSI. This is the first report of this mutation in brain tumors. *MSH6* poly(C)9 leads to a truncated protein and is probably the cause of the total absence of protein assessed by immunohistochemistry. *MSH6* mutations in the poly(C)8 microsatellite are thought to arise due to germline mutations of the MMR genes, making of *MSH6* a target for somatic mutations in the presence of MMR germline mutations [35]. These germline mutations usually occur in the context of familial tumors and accordingly this patient had a clinical history consistent with a MMR deficiency syndrome such as Turcot, in which Type A MSI has been reported in affected children with glioblastoma [44]. Recently, *MSH6* mutations have been demonstrated to arise in gliomas as a consequence of treatment with temozolomide, and have been implicated in drug resistance and the presence of a hypermutated phenotype [4], [45]. Apart from the sample RMH2458, which did not harbor any *MSH6* mutation, the tumors analyzed in this study, including the *MSH6* mutated RMH2452, were primary tumors, and have not been previously exposed to radio or chemotherapy treatment. Therefore MSH6 findings are not related with a previous exposure to temozolomide.

A major consequence of MSI is the accumulation of additional mutations in key oncogenic target genes. To our knowledge, only five known MSI target genes have been screened, with mutations reported in a single case each of *IGFIIR* [7] and *PTEN* [9], and of *TGFβRII* in 71% cases [14], although this latter observation was not seen elsewhere [7], [9]. We investigated the mutational status of 18 classical target genes in MSI HGG and, apart from *MSH6*, found alterations only in two other target genes, *DNAPKcs* and *MRE11* which are involved in DNA DSB repair. The *DNAPKcs* poly(A)9/10 sequence variant has been described in samples of gastric and endometrial tumors [46], [47], but has not been previously reported in gliomas. In subclones of the CRC cell line HCT-8 it failed to confer additional deficiency to DNA DSB repair compared to the parental line [48], and we found no alterations in protein expression in the MSI glioma sample presenting this mutation. *MRE11* poly(T)11/12 has previously been reported in a single case of MSI CRC [49], a single case of MSI medulloblastoma [21], and in the Daudi lymphoma cells [26]. Our findings here demonstrate that it appears to be an undocumented polymorphism with no functional consequences on DNA damage detection and repair. Thus it seems that the classical target genes for MSI in other tumor types are not frequently mutated in gliomas, and the field would benefit from a bioinformatic approach focusing on specific repeat sequences in coding regions for identifying novel, possibly glioma-specific MSI target genes as has been carried out in other tumor types [50].

The presence of MSI in pediatric HGG may have important translational implications. Specifically, in CRC, MSI-positive patients appear to show a differential response to 5-fluorouracil alone [51] as well as adjuvant therapy with irinotecan, fluorouracil, and leucovorin [52]. Furthermore, the majority of MSI-positive tumor cell lines of different tissue origins (endometrial, ovarian, prostate, and colorectal carcinomas) appear hypersensitive to drugs that produce DNA DSBs such as bleomycin [48]. Given the considerable impact of abrogated DNA repair capacity on gliomagenesis, identification of even a subset of cases with a differential response to existing chemotherapeutics would be of immense clinical benefit in these extraordinarily treatment refractory tumors.

In conclusion, we identified a subset of glioma patients presenting MSI with molecular alterations distinctive of this phenotype suggesting an association of MSI with the development of gliomas.

## Supporting Information

Table S1 **Clinicopathological characteristics and microsatellite screening data of all HGG samples.** Full QMVR range is provided.
(TIFF)

Figure S1 **Immunohistochemistry of MMR proteins in MSI-positive samples.** H&E staining as well as expression of MLH1, MSH2, MSH6 and PMS2 are shown for cases RMH2452, RMH2458 and RMH4816. Original magnification ×200 (inset ×600).
(TIFF)

Figure S2 **Immunofluorescence for γH2AX foci in Daudi, Jurkat and Raji cells.** Cells were treated with 1.5 Gy IR and allowed to recover for 1 h and 4 h. Higher background levels of DNA damage were observed in Raji cells as seen by the formation of foci in the non-irradiated cells.
(XLS)
