# Supplementary figures and images for: Microsatellite Instability in Pediatric High Grade Glioma Is Associated with Genomic Profile and Differential Target Gene Inactivation
Source: PLoS One. 2011 May 26;6(5):e20588. doi: 10.1371/journal.pone.0020588 (PMC3102740; doi:10.1371/journal.pone.0020588)

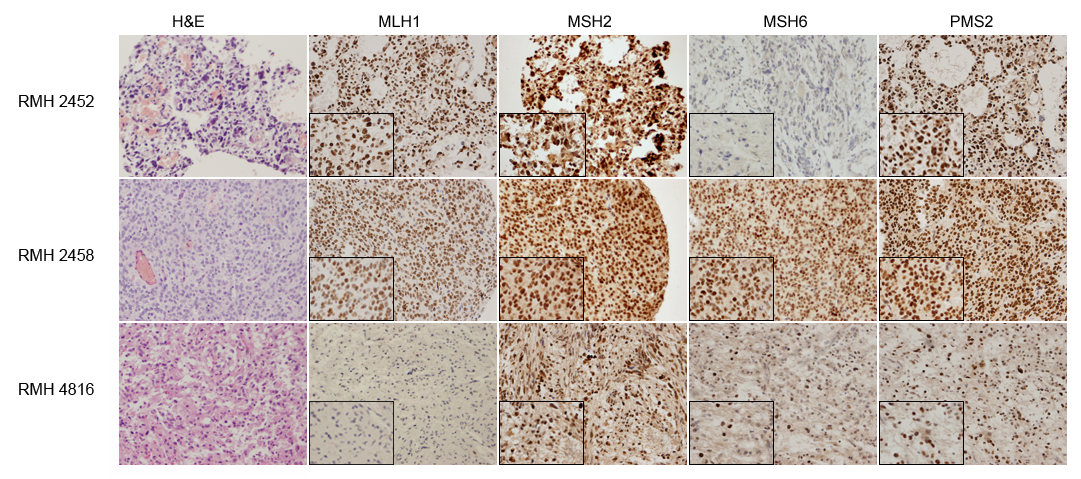

Supplement: Figure S1 — Immunohistochemistry of MMR proteins in MSI-positive samples. H&E staining as well as expression of MLH1, MSH2, MSH6 and PMS2 are shown for cases RMH2452, RMH2458 and RMH4816. Original magnification ×200 (inset ×600). (TIFF) [file pone.0020588.s002.tiff]

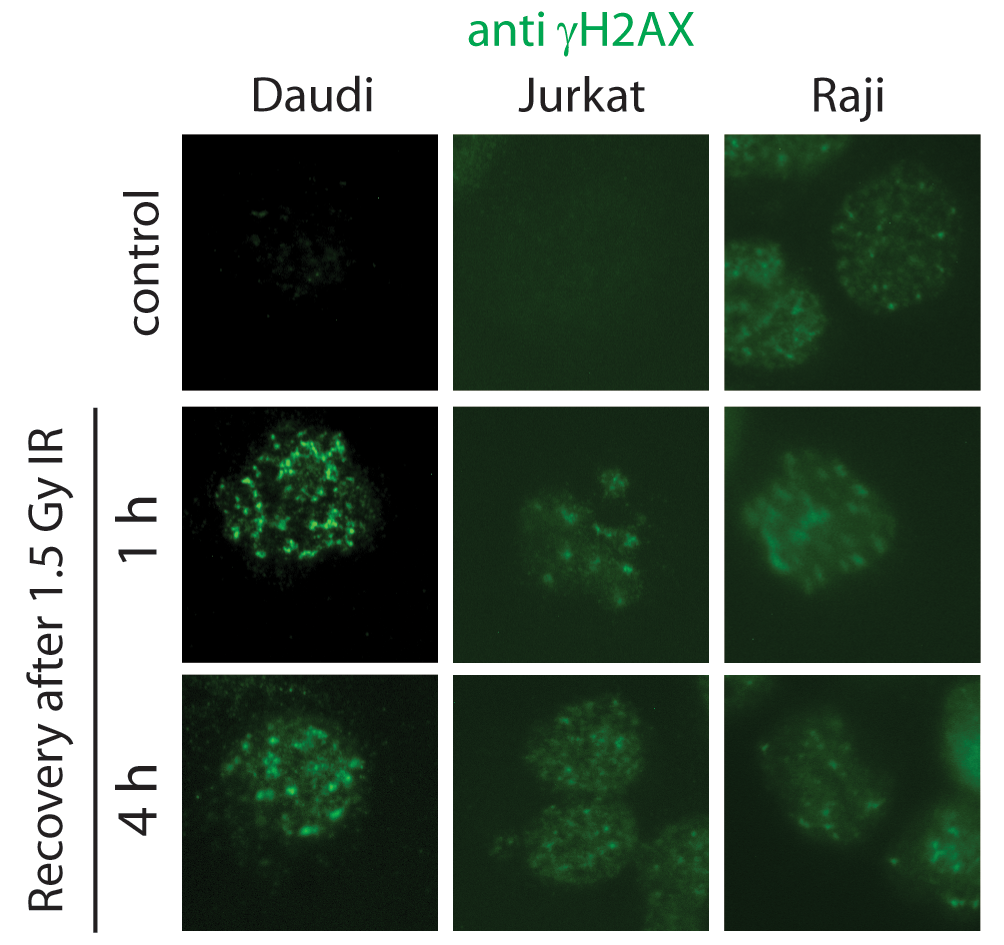

Supplement: Figure S2 — Immunofluorescence for γH2AX foci in Daudi, Jurkat and Raji cells. Cells were treated with 1.5 Gy IR and allowed to recover for 1 h and 4 h. Higher background levels of DNA damage were observed in Raji cells as seen by the formation of foci in the non-irradiated cells. (XLS) [file pone.0020588.s003.xls]
